# A pattern of antibiotic drug resistance of *Salmonella* Typhi and *Salmonella* Paratyphi among children with enteric fever in a tertiary care hospital in Lahore, Pakistan

**DOI:** 10.3325/cmj.2023.64.256

**Published:** 2023-08

**Authors:** Usman Baig, Syed Muslim Mehdi, Nosheen Iftikhar

**Affiliations:** Department of Pediatrics, Sharif Medical City Hospital, Lahore, Pakistan

## Abstract

**Aim:**

To establish the pattern of antibiotic resistance and assess the frequency of multidrug-resistant (MDR) and extensively drug-resistant (XDR) strains of *Salmonella* Typhi and *Salmonella* Paratyphi among children with enteric fever.

**Methods:**

This cross-sectional study was carried out in the Department of Pediatrics, Sharif Medical City Hospital, Lahore, from July 2020 to January 2021. The study involved patients aged between 0 to 15 years who attended our outpatient department or were admitted to the ward with the suspicion of typhoid fever. A convenience sample of patients with blood cultures positive for *S*. Typhi and *S*. Paratyphi was enrolled.

**Results:**

Of the 105 participants, 70 (66.7%) were male. The mean age was 8.48 ± 4.18 years, and the most affected age group was 6-10 years (n = 46, 43.8%). Among the cultured organisms, 95 (90.5%) isolates were *S*. Typhi and 10 (9.5%) were *S*. Paratyphi A. Antibiotic resistance was highest against ampicillin (n = 91, 86.7%), and all of the isolates were sensitive to imipenem and meropenem. Twenty-three (21.9%) cultured organisms were MDR and 54 (56.8%) were XDR.

**Conclusion:**

An alarming antibiotic drug resistance pattern was observed among children with enteric fever in Lahore. The lowest resistance was noted for azithromycin, meropenem, and imipenem. Our findings warrant the immediate implementation of tailored antibiotic stewardship and infection control strategies.

*Salmonella enterica* serovar Typhi (*S*. Typhi) and *Salmonella enterica* serovar Paratyphi (*S*. Paratyphi) are rod-shaped, gram-negative bacteria that belong to the family of enterobacteriacae (1). *S*. Typhi is the causative agent of typhoid fever, while *S*. Paratyphi serotypes A, B, and C cause paratyphoid fever. These two types of fever have indistinguishable clinical symptoms. Hence, the term “enteric fever” is used for both types of fever, and both strains of bacteria are sometimes referred to as typhoid *Salmonella* (2). Each year, about 21 million people worldwide are affected with enteric fever, of whom more than 150 000 die (3). Pakistan, a lower middle-income country, has an annual incidence of 493.5 per 100 000 persons. This rate places it among the countries significantly affected by typhoid. The residents of Punjab and Sindh provinces are at the highest risk of acquiring the infection (4,5).

Enteric fever occurs as a result of ingestion of a causative organism through consumption of food and water contaminated by fecal material. The incubation period is usually 9.7 to 21.2 days, depending on the infecting dose (6). The clinical picture can range from a moderate disease with a low-grade fever, malaise, and mild, dry cough to a severe illness with abdominal discomfort and many sequelae. The severity and clinical prognosis are influenced by a variety of factors: illness duration before the initiation of appropriate therapy, choice of antimicrobial treatment, age, previous exposure or immunization history, virulence of the bacterial strain, quantity of inoculum ingested, and immune status of the host (7).

Traditionally, the first-line treatment of enteric fever included ampicillin, chloramphenicol, and trimethoprim/sulfamethoxazole. However, due to the emergence of resistant bacteria these drugs are no longer effective. Moreover, their use led to the emergence of a multidrug-resistant (MDR) strain, which is resistant to the mentioned drugs. The MDR strain was first reported in 1970, but resistance to the first-line drugs became prevalent between 1980 and 1990 (8). This was followed by a change in the prescription trend to quinolones and cephalosporins. These drugs proved to be effective till the emergence of an extensively drug-resistant (XDR) strain, which was also resistant to quinolones and cephalosporins (9). The first outbreak of XDR typhoid occurred in Hyderabad in 2016, and it rapidly spread to other cities in Sindh province. Since then, over 10 000 cases have been reported in Pakistan alone, a number that indicates the alarming extent of the spread of this highly resistant strain (10,11).

The consequences of this problem extend beyond the borders of Pakistan. The identification of the dominant XDR genotype (4.3.1.1.P1) within three years of its first recognition in Pakistan raises concerns about the potential for sporadic cases and outbreaks with limited treatment options on a global scale (12). Given the extensive use of air travel, the cross-border spread of antibiotic-resistant bacteria becomes a plausible scenario. Notably, sporadic propagation of the drug-resistant strains has been reported in the United Kingdom and the United States (13). In light of the increase in drug resistance in the last decade, we aimed to establish the current antimicrobial drug susceptibility pattern in a hospital in Lahore.

## Methods

This descriptive cross-sectional study was carried out in the Department of Pediatrics, Sharif Medical City Hospital, Lahore, from July, 2020 to January, 2021. A required sample size of 105 was calculated by using the WINPEPI statistical program (http://www.brixtonhealth.com/pepi4windows.html), with a confidence of 95%, acceptance difference of 0.06, and an assumed proportion of 0.89 (14,15). The targeted population were patients aged between 0 to 15 years who attended the outpatient department or were admitted to the ward with a suspicion of typhoid fever. A convenience sample of patients with blood cultures positive for *S*. Typhi or *S*. Paratyphi was enrolled. The parents of all the included patients provided informed consent. The study was approved by the Ethics Committee of Sharif Medical and Dental College, Lahore (SMDC/SMRC/115-20).

Data on baseline socio-demographic characteristics were obtained (Table 1). Five milliliters of venous blood were drawn by well-qualified paramedical staff using aseptic techniques and sent to the laboratory for culture and sensitivity testing. The blood samples were dispensed in pediatric blood culture bottles (Medilines, Lahore, Pakistan) and incubated for 24 hours. The first sub-culture was conducted on day 1 on MacConkey agar and Blood agar with in-house prepared culture media (Oxoid, Basingstroke, UK). The specimens showing positive growth were sent for identification by Gram staining and API20E, and for antibiotic sensitivity testing. A second subculture was conducted on day 5, and specimens with positive growth were sent for identification and antibiotic sensitivity testing. Samples not showing growth were reported negative after seven days of incubation. Antimicrobial resistance against the following groups of antibiotics was tested: penicillins (ampicillin and amoxicillin clavulanate), sulphonamides (trimethoprim/sulfamethoxazole), fluoroquinolones (ciprofloxacin and levofloxacin), cephalosporins (ceftriaxone and cefixime), macrolides (azithromycin), carbapenems (imipenem and meropenem), and chloramphenicol. Isolates were divided into two groups: MDR and XDR. Antibiotic disc (Oxoid) potencies of the listed antibiotics are shown in Table 2. Clinical and Laboratory Standards Institute guidelines were used to interpret the results (16).

**Table 1:** Participants’ sociodemographic characteristics

	Frequency (n)	Percentage (%)
Age group (years)		
1-5	26	24.8
6-10	46	43.8
11-15	33	31.4
Sex		
male	70	66.7
female	35	33.3
Type of family		
nuclear	43	41.0
extended	62	59.0
Education level (of the mother)		
illiterate	15	14.3
literate		
grade 1-5	19	18.1
grade 6-8	24	22.9
grade 9-10	12	11.4
grade 11-12	20	19.0
college or higher	15	13.3
Education level (of the father)		
illiterate	11	10.5
literate		
grade 1-5	13	12.4
grade 6-8	16	15.2
grade 9-10	22	21.0
grade 11-12	25	23.8
college or higher	18	17.1
Total family income (in PKR)		
below or at minimum wage (<30 000)	12	11.4
middle income (30 000-50 000)	25	23.8
higher income (>50 000)	68	64.8
Drinking water		
tap water/unboiled	52	49.5
boiled water	31	29.5
mineral water	22	21.0
Outside food consumed in last month (days)		
0	17	16.2
1-10	51	48.6
11-20	24	22.8
21-30	13	12.4

**Table 2:** Antibiotic sensitivity trend in isolates of *Salmonella* Typhi and *Salmonella* Paratyphi A

Antibiotics	Disc potency (μg)	Bacterial strains	Antibiotic susceptibility		p
			sensitive n (%)	resistant n (%)	
Ampicillin	10	*S*. Typhi	8 (8.42)	87 (91.58)	<0.001
		*S*. Paratyphi A	6 (60.0)	4 (40.0)	
Amoxicillin clavulanate	30	*S*. Typhi	45 (47.37)	50 (52.63)	0.201
		*S*. Paratyphi A	7 (70.0)	3 (30.0)	
Trimethoprim/sulfamethoxazole	25	*S*. Typhi	16 (16.84)	79 (83.16)	<0.001
		*S*. Paratyphi A	9 (90.0)	1 (10.0)	
Chloramphenicol	30	*S*. Typhi	10 (10.52)	85 (89.48)	0.001
		*S*. Paratyphi A	6 (60.0)	4 (40.0)	
Ciprofloxacin	5	*S*. Typhi	14 (14.74)	81 (85.26)	0.003
		*S*. Paratyphi A	6 (60.0)	4 (40.0)	
Levofloxacin	5	*S*. Typhi	18 (18.95)	77 (81.05)	<0.001
		*S*. Paratyphi A	10 (100.0)	0 (0.0)	
Ceftriaxone	30	*S*. Typhi	36 (37.89)	59 (62.11)	<0.001
		*S*. Paratyphi A	10 (100.0)	0	
Cefixime	5	*S*. Typhi	30 (31.58)	65 (64.82)	<0.001
		*S*. Paratyphi A	10 (100.0)	0 (0)	
Azithromycin	15	*S*. Typhi	82 (86.32)	13 (13.68)	0.357
		*S*. Paratyphi A	10 (100.0)	0 (0)	
Imipenem	10	*S*. Typhi	95 (100.0)	-	-
		*S*. Paratyphi A	10 (100.0)	-	
Meropenem	10	*S*. Typhi	95 (100.0)	-	-
		*S*. Paratyphi A	10 (100.0)	-	

### Statistical analysis

Sociodemographic characteristics and antibiotic sensitivity trends are presented as frequencies and percentages. The significance of differences between the groups was assessed with a post-stratification Fisher exact test. *P* ≤ 0.01 was considered statistically significant. The analysis was conducted with SPSS, version 23 (IBM Corp, Armonk, NY, USA).

## Results

The study enrolled 105 participants (66.7% boys), with a mean age of 8.48 ± 4.18 years. The most affected age group was 6-10 years (43.8%). Most participants lived with the extended family (59.0%), had a total family income greater than PKR 50 000 (64.8%), and relied on tap water (49.5%). Almost half of the participants (48.6%) had consumed food prepared outside home in the last 1-10 days (Table 1).

Among the cultured organisms, 95 (90.5%) isolates were *S*. Typhi and 10 (9.5%) were *S*. Paratyphi. Drug resistance was highest against ampicillin (n = 91, 86.7%) and was zero against imepenem and meropenem (Table 2). Seventy-seven (73.3%) isolates were resistant to first-line antibiotics. The susceptibility to azithromycin and amoxicillin clavulanate was similar between *S*. Typhi and *S*. Paratyphi (*P* = 0.357 and *P* = 0.201, respectively).

Twenty-three (21.9%) cultured organisms were MDR, of which 22 (95.7%) were *S*. Typhi. Fifty-four (56.8%) were XDR, all of them being *S*. Typhi (*P* < 0.001) (Table 3). A considerable number of XDR *S*. Typhi strains (25/44, 56.8%) was observed in participants who had consumed food prepared outside home in the last 1-10 days (*P* = 0.01). Similarly, a higher number of strains resistant to first-line antibiotics (n = 43/45, 95.6%) was observed in participants who used unboiled/tap water (*P* < 0.001). This pattern was also present among participants with XDR strains: 29 out of 45 participants with XDR strains (64.4%) reported the use of unboiled/tap water (*P* = 0.02).

**Table 3:** Multidrug resistant (MDR) and extensively drug resistant (XDR) strains in cultured isolates

	MDR		*P* Value	XDR		*P* Value
	positive n (%)	negative n (%)	0.68	positive n (%)	negative n (%)	<0.001
***S.* Typhi**	22 (23.1)	73 (76.8)	54 (56.8)	41 (43.2)
***S.* Paratyphi A**	1 (10.0)	9 (90.0)	0 (0)	10 (100)

*Fisher exact test.

The resistance patterns of *S*. Typhi strains were determined against three different combinations: first-line antibiotics and azithromycin; ceftriaxone and azithromycin; as well as cefixime and azithromycin. Only 5 (6.1%) of the *S*. Typhi strains demonstrated sensitivity to both first-line antibiotics and azithromycin, while a significant majority of 90 (94.7%) showed resistance to at least one of the first-line antibiotics and azithromycin. Among these antibiotics, azithromycin exhibited the lowest resistance rate, with resistance observed in only 13 (13.7%) participants (*P* < 0.001).

**Figure 1:**
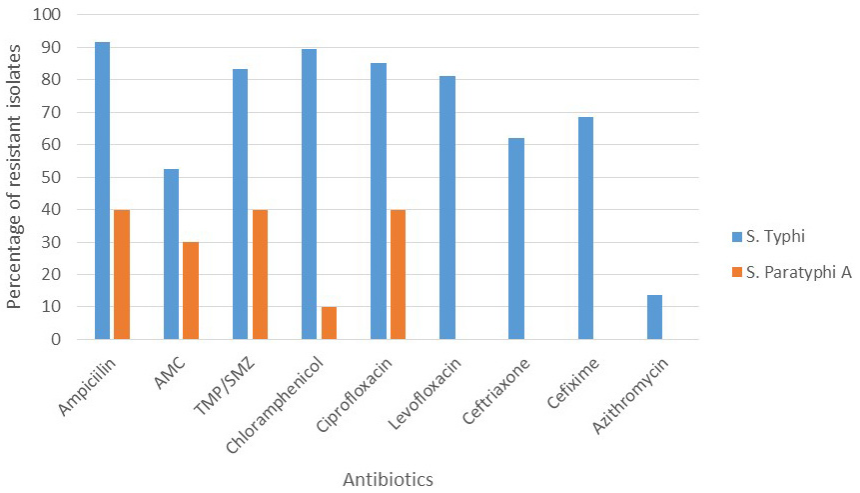
Antimicrobial resistance in the isolates of *Salmonella* Typhi and *Salmonella* Paratyphi A. AMC - amoxicillin clavulanate; TMP/SMZ - trimethoprim/sulfamethoxazole.

Regarding the combination of ceftriaxone and azithromycin, 32 (33.7%) *S*. Typhi isolates were sensitive to both antibiotics. Fifty-four (56.8%) were resistant to one of them and 9 (9.5%) were resistant to both. Among the isolates resistant to ceftriaxone, 50 (84.7%) demonstrated sensitivity to azithromycin (*P* < 0.001). Similarly, 26 (27.4%) of the *S*. Typhi isolates were sensitive to both cefixime and azithromycin, whereas 60 (63.2%) were resistant to one of them. Nine (9.5%) strains were resistant to both cefixime and azithromycin. Among the isolates resistant to cefixime, 56 (86.2%) demonstrated sensitivity to azithromycin (*P* < 0.001). The overall resistance to antibiotics was significantly lower in *S*. Paratyphi than in *S*. Typhi (Figure 1).

## Discussion

This study highlighted an alarming pattern of antibiotic drug resistance among children with enteric fever in Lahore. The frequency of enteric fever was greater in schoolchildren (aged 6-15 years), compared with pre-school children. A similar a result was found in a study conducted in Delhi (17). This finding could be explained by poor eating habits, a lack of sanitation and frequent exposure to contaminated food, particularly in schoolchildren. In the current study, enteric fever was observed more frequently in boys, participants who relied on tap water consumption, and those who had consumed food prepared outside of home. Research conducted in Hyderabad observed the same risk factors (18). These findings suggest that infection is attributable to short-cycle transmission, which involves direct contamination via the feco-oral pathway. Mobile street vendors and corner stores could play an important role in this context (11,19). They frequently place their carts near drainage systems and overlook essential hygiene procedures, such as handwashing during food preparation and raw goods handling. Similarly, corner stores use unregulated food sources, display food without proper protection, lack sanitation on food preparation surfaces, and lack proper refrigeration. Another source of infection could have been the use of unboiled/tap water, which the majority of participants relied on. This type of water is not tested and is frequently contaminated with bacteria above the safe consumption level.

The antibiotic resistance observed in this study is significantly higher than that reported in the national antibiotic resistance monitoring systems of the United States and the European Centre for Disease Prevention and Control (20,21). While in these countries, the greatest resistance was observed against ciprofloxacin, we observed the greatest resistance against ampicillin, but the level of ciprofloxacin resistance was higher than in the mentioned countries. The differences could be attributed to antibiotic stewardship programs, a well-developed health care infrastructure, and an exceptional surveillance and monitoring system in these countries. In contrast, Pakistan, a country facing economic challenges, can only allocate less than USD $200 million for its entire health care system (19).

The rate of *S*. Paratyphi isolates in our study was lower than that observed in other studies, a difference attributable to regional differences (22,23). In line with studies conducted in India, the resistance to amoxicillin clavulanate was lower than that to ceftriaxone and first-line antibiotics (24,25). The resistance to ampicillin, chloramphenicol, and ciprofloxacin, and the sensitivity to azithromycin, meropenem, and imepenem was similar to that in other studies conducted in Pakistan (15,26,27). The percentage of isolates resistant to first-line antibiotics was also comparable to the findings of a recent study conducted in Karachi (27). The trend of antibiotic resistance clearly indicates a continuous increase when compared with studies conducted in the past decade (28). This increase can largely be attributed to injudicious use of antibiotics, which includes self-medication, the prescription of inappropriate antibiotics by unqualified practitioners, and easy access to various types of antibiotics at pharmacies without a valid prescription.

A factor contributing to improper administration of medications is the presence of over 50 000 clinics managed by unqualified medical professionals. In contrast to qualified general practitioners, quacks do not charge consultation fees, a practice that appeals to the underprivileged and less educated population. The government has responded to the urgency by closing over 20 000 of these clinics. In addition, Islamabad, the capital of Pakistan, has implemented a policy to regulate the sale of antibiotics. However, for these measures to effectively tackle the deteriorating antibiotic resistance, they must be implemented nationwide (29).

In this study, one-third of the *S*. Typhi isolates were sensitive to ceftriaxone/cefixime and azithromycin. This combination therapy is the standard approach in Sheba Medical Center, Israel, and is currently being investigated in a phase-IV randomized controlled trial expected to be completed by April 2024 (30-32). The combination seems to be especially potent as azithromycin exerts maximum effects intracellularly and ceftriaxone/cefixime exerts its effects extracellularly. Additionally, our data suggest that azithromycin could be a valuable addition to ceftriaxone/cefixime in empirical treatment in areas where ceftriaxone resistance is common and would also delay emergence of resistance. There is currently only one clinical trial on the combination of ceftriaxone and azithromycin for the treatment of enteric fever, conducted in Nepal; however, the study is limited by a small sample size (32). Further research and results from the ongoing clinical trial are required to assess the clinical response, treatment outcomes, and safety of this therapeutic regimen.

The prevalence of the MDR strain ranges from 10%-80% in endemic areas, a rate that agrees with our findings (33-35). The prevalence of the XDR strain was also similar to other studies conducted in Pakistan. This is an alarming finding indicating a threat to health care system (26,27). A study from Karachi showed lower percentages of MDR and XDR isolates, which could be explained by a lower antibiotic resistance in that particular area (36). The prevalence of MDR bacteria is decreasing in India and Bangladesh, accompanied by an increased sensitivity to first-line antibiotics, whereas MDR strains are still being reported in Pakistan and Nepal (37,38). This decline may be attributed to their low prescription rates. A higher resistance to ciprofloxacin in India compared with our study could be due to its excessive use.

In our hospital, the first-line antibiotics used to treat suspected enteric fever are either oral cefixime or intravenous ceftriaxone. We consider adding azithromycin to the regimen if patients show a partial or no response to the initial treatment, or if they had been taking antibiotics before presentation. The decision to escalate or de-escalate treatment after obtaining antimicrobial susceptibility testing results is made on a case-by-case basis, taking into account the patient's clinical response and the susceptibility profile of the isolated pathogen. We observed ceftriaxone resistance in 59 patients and therefore escalated the treatment regimen to alternative antibiotics such as azithromycin or meropenem. This finding highlights the need for multidisciplinary collaboration among clinicians and microbiologists to guide appropriate therapy based on resistance patterns, as well as the importance of antibiotic stewardship in managing enteric fever in our hospital. As the national antibiotic stewardship program is still being developed, the antibiotic stewardship program of our hospital specifically reserves the use of azithromycin for treating salmonella infections, purposefully avoiding its administration for respiratory indications to mitigate the risk of antibiotic resistance development.

Until proper reforms are made in the health care infrastructure, vaccines offer an effective short-term solution to the spread of typhoid and the alarming level of antibiotic resistance. In 2019, Pakistan became the first country to include typhoid vaccination (at the age of nine months) in the extended immunization program. A two-week mass vaccination campaign was launched in Sindh province in 2019, followed by similar campaigns in other provinces in 2021 and 2022 (19,39,40). Despite these efforts, vaccination rates in rural areas are poor due to cultural and religious stigmas associated with vaccines. Some of these beliefs are that vaccinations include ingredients prohibited by Islamic law and that vaccines are part of an international conspiracy against Pakistan (19,39). Similar beliefs were related to other vaccines as well, including the belief that polio and COVID-19 vaccines cause infertility. These misconceptions should be addressed by awareness campaigns to increase vaccination rates in rural areas.

The limitations of the current study include the fact that the study was restricted to a single hospital in Lahore, a small sample size, and a lack of genotyping.

The findings of this study could be used to inform pediatricians on the adequate management of enteric fever and selection of appropriate antibiotics. They also contribute to the growing body of knowledge on drug resistance and can support the efforts to combat the spread of MDR strains. The high prevalence of strains resistant to a wide range of antibiotics, coupled with the emergence of MDR and XDR strains, underscores the need for targeted antibiotic stewardship protocols and comprehensive infection control strategies in the city.
